# Apothecaries

**Published:** 1847-10

**Authors:** 


3. *To the Editor of the Annalist: Sir,*—It is a matter of surprise to me, as I know it is to yourself, that so little interest is manifested by the profession at large in reference to the exposure of quackery, and many kindred evils which operate so injuriously to their interests. One of these kindred evils I wish to bring to your notice, and through your valuable periodical to introduce the same to the particuiar attention of my professional brethren. I mean the very general practice of our apothecaries in prescribing for, and administering to, individuals who choose to consult them. This is an evil of which all have reason to complain, and it is one which I think the Academy of Medicine would do well to notice, with the view of taking certain steps towards its removal. Go into any apothecary's shop you please, and remain there half an hour or so, and you will almost always find some one come in to consult the apothecary, who never lets him go away without a dose of some sort. This is a common practice among the fraternity, and is one which is fraught with serious evils; for these persons are not always competent to prescribe, if they are to compound. Not unfrequently great mistakes are made by them, in prescribing either the wrong remedy, or in giving an over dose, and death may be the consequence. The apothecary has no just right thus to interfere with what strictly belongs to the physician. His business is to provide himself with the purest drugs, and to compound them according to the prescribed formulæ. It is not necessary for him to be thoroughly acquainted with their particular properties, doses, &c., though some knowledge of this kind is useful to him, as well as to the physician who employs him.

I hope that, as the profession has of late manifested a disposition to maintain its dignity, and to advance its interests by a bold and decided opposition to whatever bear the least relation to quackery, it will see the propriety—nay, the necessity of adopting some measures against this encroachment on one of its principal prerogatives.

Let but unanimity of sentiment and unity of action prevail, and in due season the object will be accomplished.

MEDICUS.

